# School inclusion of children and adolescents with epidermolysis bullosa: The mothers’ perspective

**DOI:** 10.1590/1980-220X-REEUSP-2022-0271en

**Published:** 2022-11-28

**Authors:** Nayara Gonçalves Barbosa, Carolina Balestra Silva, Diene Monique Carlos, Lilian Brosso, Aline Fernanda Levada, Aline Cristiane Cavicchioli Okido

**Affiliations:** 1 Universidade Federal de Juiz de Fora, Faculdade de Enfermagem, Departamento de Enfermagem Materno-Infantil e Saúde Pública, Juiz de Fora, MG, Brazil.; 2 Universidade Federal de São Carlos, Curso de Especialização em Enfermagem Pediátrica, São Carlos, SP, Brazil.; 3 Universidade de São Paulo, Escola de Enfermagem de Ribeirão Preto, Ribeirão Preto, SP, Brazil.; 4 Universidade Federal de São Carlos, Programa de Pós-Graduação em Enfermagem, São Carlos, SP, Brazil.

**Keywords:** Epidermolysis Bullosa, Mainstreaming, Education, Nursing Care, Child Health, Chronic Disease, Epidermólisis Ampollosa, Integración Escolar, Atención de Enfermería, Salud Infantil, Enfermedad Crónica, Epidermólise Bolhosa, Inclusão Escolar, Cuidados de Enfermagem, Saúde da Criança, Doença Crônica

## Abstract

**Objective:**

to understand the school inclusion of children and adolescents with Epidermolysis Bullosa from the mothers’ perspective.

**Method:**

qualitative study, based on Urie Bronfenbrenner’s Bioecological Theory of Development, conducted between September and November 2021. Interviews were conducted with six mothers from different Brazilian locations, recording audio and video using the Google Meet® platform. The statements were analyzed using thematic analysis.

**Results:**

two categories were identified: i) The school microsystem: challenges and adaptations for inclusion of children and adolescents with Epidermolysis Bullosa; ii) The school-family mesosystem: possibilities to promote better school inclusion. Mothers highlighted the challenges in school inclusion as well as the benefits provided by social interaction. In order to facilitate the inclusion, the school microsystem promoted adaptations in the teaching-learning process, structural changes, hiring of caregivers, and dialogues with family members.

**Conclusion:**

initially, school inclusion was permeated by feelings such as fear and anguish, but the adaptations contributed to promote well-being, welcoming, and social integration of children and adolescents.

## RESUMO

**Objetivo:**

compreender a inclusão escolar de crianças e adolescentes com epidermólise bolhosa na perspectiva materna.

**Método:**

estudo qualitativo, fundamentado na Teoria Bioecológica do Desenvolvimento de Urie Bronfenbrenner, realizado entre setembro e novembro de 2021. Foram realizadas entrevistas com seis mães de diferentes localidades brasileiras, áudio e vídeogravadas, utilizando a plataforma Google Meet®. Os depoimentos foram analisados mediantes análise temática.

**Resultados:**

Foram identificadas duas categorias: i) O microssistema escolar: desafios e adaptações para inclusão de crianças e adolescentes com epidermólise bolhosa; ii) O mesossistema família-escola: possibilidades para promover maior inclusão escolar. As mães destacaram os desafios da inclusão escolar, bem como os benefícios proporcionados pela interação social. A fim de facilitar a inclusão, o microssistema escolar promove adaptações do processo de ensino aprendizagem, mudanças estruturais, contratação de cuidadores e interlocução com os familiares.

**Conclusão:**

A inclusão escolar foi inicialmente permeada por sentimentos como medo e angústia, mas as adaptações contribuíram para promover bem-estar, acolhimento e integração social das crianças e adolescentes.

## RESUMEN

**Objetivo:**

estudiar la inclusión escolar de niños y adolescentes con Epidermólisis Bullosa desde la perspectiva materna.

**Método:**

estudio cualitativo, fundamentado en la Teoría Bioecológica del Desarrollo de Urie Bronfenbrenner, realizado entre septiembre y noviembre de 2021. Se realizaron entrevistas a seis madres de diferentes localidades brasileñas, grabadas en audio y vídeo, utilizando la plataforma Google Meet®. Las declaraciones se analizaron mediante un análisis temático.

**Resultados:**

surgieron dos categorías: i) El microsistema escolar: desafíos y adaptaciones para la inclusión de niños y adolescentes con Epidermólisis Bullosa; ii) El mesosistema escuela-familia: posibilidades de promover una mejor inclusión escolar. Las madres destacaron los retos de la inclusión escolar, así como los beneficios que aporta la interacción social. Para facilitar la inclusión, el microsistema escolar promovió adaptaciones del proceso de enseñanza-aprendizaje, cambios estructurales, contratación de cuidadores y diálogo con los familiares.

**Conclusión:**

al comienzo la inclusión escolar estaba impregnada de sentimientos como miedo y angustia, pero las adaptaciones contribuyeron a promover el bienestar, la acogida y la integración social de los niños y adolescentes.

## INTRODUCTION

Epidermolysis Bullosa (EB) is a hereditary condition, part of a heterogeneous group of rare genodermatoses characterized by fragility of the skin and mucous membranes with blister formation at minimal trauma, besides the occurrence of cutaneous erosions and ulcerations^(1, 2)^. More than 30 subtypes of the disease are recognized, classified into four major categories according to the level of cleavage and its clinical and molecular characteristics: simple EB, junctional EB, dystrophic EB and Kindler syndrome^(1–3)^.

It is estimated that approximately 500,000 people worldwide are carriers of EB^(3)^. In the United States, the prevalence of EB in the population is 11.1 per 1,000,000 and its incidence is 19.6 cases per 1,000,000 live births^(2)^. In Brazil, there is no concrete dimension about the epidemiological data of EB, since it is a rare condition and it is not part of the list of diseases and diseases of compulsory notification^(4)^.

Skin lesions demand permanent care and observation^(3)^ and dressing is generally a painful and slow process that can last several hours each day^(1)^. Frequent and larger lesions impact the performance of activities of daily living^(3, 5)^ such as sitting, writing and playing, significantly affecting the child and adolescent’s daily life^(5)^. Therefore, children and adolescents with EB represent a subgroup of Children with Special Health Care Needs (CSHCN), a broad definition that encompasses different conditions that vary in complexity and care demands, having as a common characteristic the need for continuous attention from family members and health professionals, beyond what is required by children of the same age group^(6)^.

Life expectancy is not affected in most forms of EB^(1)^, especially in those with moderate manifestations^(7)^. However, EB patients have lower life satisfaction^(3)^, numerous physical and emotional traumas and anxiety^(5)^. Added to the precedent it must be taken into account what they represent as a burden for caregivers^(3)^. The demand for care associated with health needs, the pain and discomfort inherent to this condition have a significant impact on the life of the person and family members. Consequently, there are implications in the relational interactions of family, friends, and peers, also influencing the issues of employment, leisure, and education^(1)^.

In this sense, the needed adaptations for school inclusion of children and adolescents with EB represent a major challenge, in ensuring the constitutional right to equal conditions for access and permanence in school, without any kind of discrimination^(8)^. Regarding the school inclusion of children and adolescents with EB, there are numerous challenges including the consideration of the child’s clinical conditions related to skin fragility, pain, the presence of dressings and mobility limitations^(3, 9)^, in addition to the relational interactions with other children and adolescents, parents and teachers, considering the stigma of the disease and the lack of knowledge in the school environment^(5)^, resulting in the isolation of children/adolescents with EB and even the experience of bullying^(3, 7, 10)^.

Facing the complexity of demands of CSHCN requires the integration of nurses with the social network formed by institutional, family and community participation, increasing the visibility of the demands of care and articulating them to the different contexts related to the care of CSHCN^(11)^, even in the process of school inclusion. Thus, nurses contribute to the protection and promotion of the rights of this population to inclusion in health, education and social care services, among others, in order to strengthen actions that promote the development of these children, a fundamental condition for an inclusive society^(12)^.

Considering the above, the object of this study is the school inclusion of children and adolescents with EB from a mothers’ perspective. There is a lack of studies in the nursing area that investigate the school inclusion of children and adolescents with EB in the Brazilian population, justifying the relevance and pertinence of the proposal. Considering the premises of the School Health Program^(13)^, established in 2007, it is important to address health vulnerabilities, such as the experience of chronic conditions that may compromise the full school and personal development. In this direction, the present study aims to understand the school inclusion of children and adolescents with EB from the mothers’ perspective.

## METHOD

This is a qualitative study^(14)^ based on the Bioecological Theory of Development, proposed by Urie Bronfenbrenner^(15)^. This perspective considers that human development occurs continuously and contextually, from a primary interactive environment with other environments, in successive and interconnected structures. These components are: individual (biological and individual characteristics of the person in development); microsystem (relationships close to the person, such as family and school); mesosystem (relationships between two microsystems in which the person is inserted); exosystem (indirect relationships between the person in development, such as family work) and macrosystem (social, historical, political and cultural aspects of the context in which the person is inserted)^(15)^. Due to the particularities of this study, the relationships between children/adolescents with EB (people in development), their school microsystem and school-family mesosystem will be emphasized. The guidelines of the Consolidated Reporting Criteria for Qualitative Research (COREQ)^(16)^ were used to ensure methodological rigor.

### Population and Selection Criteria

This is a section of a larger study that evaluated the experience of ten mothers in the care of children and adolescents with EB. Six children attended or had attended school and had experiences to share regarding the school inclusion process. Mothers of children or adolescents with EB, over 18 years old, were included. The exclusion criterion was not being the main responsible for the child’s care.

### Data Collection

Data were collected from September to November 2021. The recruitment of potential participants of the study was through the dissemination of the research on social networks (Instagram® and Facebook®) of groups or non-governmental organizations (NGOs) to support families, children and adolescents with EB. We also perused the list of followers of these groups or pages, in order to identify mothers of children/adolescents with EB who shared their daily experiences in the digital media. Based on this information, the research invitation was sent individually, by direct message to the registered profiles.

The message contained a personal introduction of the researcher, the study objectives, and an e-mail address for contact. In total, 48 mothers were invited to participate in the study, of which 14 showed interest in participating. However, four did not show up virtually on the scheduled day and time and did not answer the subsequent messages. Thus, a convenience sample of ten participants was constituted, six of whom presented experiences about the school inclusion of their children and adolescents.

After the expression of interest, one of the researchers contacted the mothers to schedule the semi-structured interview, which was held remotely and recorded on Google Meet®. The interviews were conducted by an undergraduate nursing student, through previous training, and supervised by two nurses with doctoral degrees, experienced in qualitative research. Interviews had an average length of 29 minutes and were conducted once for each participant.

The data collection instrument followed a semi-structured script composed of two parts: the first referred to the participants’ sociodemographic characterization, such as the mother’s and the child’s/adolescent’s age, marital status, income, living conditions, and the children’s school characterization (public or private school, education); the second part was composed of open questions related to the school inclusion of the child and/or adolescent with EB, as per the following: how have the child’s/adolescent’s adaptations been at school? In the classroom period, how was the experience at school and with other children/adolescents? Have you noticed any difficulties or barriers in learning? How did your child feel at school? The interviews lasted an average of 29 minutes. The interviews were conducted only once per participant.

### Data Analysis and Treatment

After collecting the data, the interviews were transcribed. The content of the interviews was ordered based on the full transcription of the recordings. To ensure the confidentiality of the participants, the interviews were identified by the letter “P”, followed by an ordinal number, according to the order in which the interviews were conducted (from P1 to P6). Fictitious names were used for the children that were mentioned during the mothers’ speeches.

The coding of the data obtained was carried out manually by two researchers, and a third researcher was consulted when there were discrepancies before the reading and coding of the data. The choice of reference framework for the analysis of the interview data comprised the Content Analysis, thematic mode, i.e., an analysis of the “meanings”, according to the phases: pre-analysis, material exploration, treatment of results, inference and interpretation^(17)^.

Two thematic categories emerged from the data analysis: i) The school microsystem: challenges and adaptations for the inclusion of children/adolescents with Epidermolysis Bullosa; ii) The school-family mesosystem: possibilities to promote better school inclusion.

### Ethical Aspects

The research was approved by the Research Ethics Committee of the Ribeirão Preto School of Nursing, University of São Paulo, with opinion number 4.952.903 of 2021. The research strictly followed the guidelines proposed by resolution 466/2012 of the National Research Council (CONEP) for research with human beings.

## RESULTS

**Chart 1.**
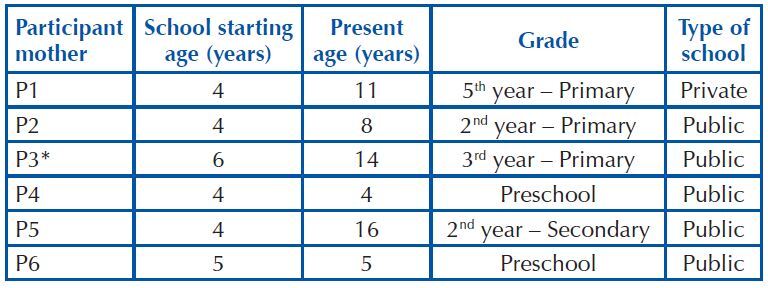
Characteristics of the school context of children and adolescents with EB who participated in the study – Brazil, 2021.

The participants were a total of six mothers of children and adolescents aged 4 to 16 years, mean age 9.66 (±4.8), predominantly male (n = 4), from the Brazilian Southeast (n = 5) and Northeast (n = 1) regions. The mean age of the women was 38.2 years (±7.9), as to skin color four were white and two were brown/black; in relation to marital status, three were divorced, three had no source of income, four had their own house, one was renting and one lived in a lent house. Chart 1 presents information about the school context of the children and adolescents with EB who participated in the study.

*At the time of the interview, the adolescent was not attending school due to the limitations of the disease, having attended school until the 3rd grade of secondary school.

***The school microsystem: challenges and adaptations for the inclusion of children/adolescents with Epidermolysis Bullosa***

The school inclusion of a child/adolescent with EB, i.e., the construction of the relationships established between this developing person and his/her school microsystem, requires a careful look and planning by the family. The first contact with the school was seen as challenging, especially because of the curious looks and silent judgments, careless speeches, gestures and attitudes about the condition of the child/adolescent with EB.

*(…) Difficult, as you know. Children are very curious. They ask, they question, you know? Curious children, sometimes prejudiced parents. Sometimes, the parents themselves say: “just don’t go near the little boy because sometimes it is contagious (…)”. If it was so, I would also have it, his father would also have it, his brothers would also have it.* (P2)

Among the existing prejudices is the judgment that these children and adolescents have cognitive or learning deficits. The mothers’ speeches brought up such perceptions veiled by the affirmation of their children’s intelligence and cognitive capacity: *He is very intelligent (…). Not because he is my son, but he is very intelligent. I noticed that he learns things very fast, he doesn’t have any difficulties at all.* (P2)

*They needed to know that she was normal, perfectly, cognitively perfect*. (P1)

However, despite the challenges, the insertion of the child with EB into the school environment represented an opportunity for integration and social enrichment, providing contact and interaction with other children.

*It’s been very good for him, because he is too attached to me and his father, he didn’t have this thing of living with other children. First, because we didn’t have time to take him to a cousin’s house, a relative’s house to play (…) the short time we have we want to rest, right? So, for him it has been wonderful, wonderful*. (P6)

The school microsystem allows a diversity of ways of socialization and integration with other children and adolescents, expanding the possibilities of healthy development of these actors:

*(…) the children want to go to the yard to run, play pique, play burn, and Valentina (fictitious name) doesn’t go down because of all this movement, but she has three little friends who keep her company (…), so, the school, for these girls, released the cell phone (…) and they play in the classroom, playing with TikTok®, playing with that, that is in fashion now, Pop It®, which is anti-stress, and they play online, so she does not feel isolated, alone in the classroom*. (P1)

*Pedro (fictitious name) is very popular, right? He closes down the school, you have no idea, the principal sometimes complained to me, she used to say: “he comes here, the girls go all over him, he is encircled by them, it’s that march (…)”.* (P5)

Another aspect revealed by the mothers concerns the functional deficit of the hand due to deformities caused by the progression of the disease, a situation that impairs writing and requires adaptations of the school microsystem:

*Although she had surgery on her hand, her hand atrophied again, so she only has her right hand, she only has the pincer movement, but then she’s tired because it forces the joint (…) it is a lot of photocopied sheets for her to just put the answer*. (P1)

*Due to the hand being atrophied, he has no coordination, he can pick up a pencil with difficulty, but he has no coordination to write. What they are using with him more is the visual even without wanting to push him too much trying to write* (P6)

The presence of a professional caregiver to help the child/adolescent with her basic needs (hygiene, food, comfort, safety) and also educational needs, such as writing, was highlighted as an important achievement:

*She needed someone, especially at this age of 4, to help her walk, take care with her own body, serve more or less as a bubble wrap for Valentina, and the school made the person available, who stayed with her from 2014 until 2020 (…) the caregiver helped her to open the case, open the snacks, to write, she kept playing the role of a scribe for her*. (P1)

*There is a person who accompanies him, to help him move around, go to the bathroom, sometimes to feed himself. (…) the school hired someone to take care of him, so if he needs to go to the bathroom, this person will take him there, go up or down a step. Very nice, he is doing super well* (P4).

Due to the fragility of the skin, small friction or traumas can cause the formation of blisters. In this sense, we observed the adaptation in the behavior of the other children in the class, in order to protect the friend with EB, denoting the importance of this microsystem for the psychosocial development of all involved. *When lining up, they were careful not to step on Valentina’s feet, mainly during recreation times, the children were very careful with her, and this class was growing together* (…) (P1).

The presence of the caregiver at school eventually conflicts with the development of autonomy, especially in the case of the adolescent’s mother’s report. It is observed the desire to break through his barriers and limitations, to explore his possibilities and to be recognized as a subject. That is, this aspect deals with a healthy adolescent development, which must be empowered by the school microsystem: *The other day they kicked a ball there and it got him, I didn’t even know what happened, he didn’t tell me, I found out because the other day the father of the boy who kicked the ball called me to apologize (…) and the ball got him in the side of his face and I asked him what happened, why did it hurt? And he said: “ah, I was taking off my blouse with everything like that and it caught and hurt” and that was it (…). He has a troop of friends who do everything, he says: “I do not need a caregiver, I do not know what”, because the person stays on top of him, right?* (P5)

It is noticed in the final excerpt the need of a balance between the support of a caregiver and the experimentation of autonomy, within a development context that enables this aspect to the adolescent. It also denotes the relevance of the school in this process.

Hot and humid environments can favor the appearance of blisters, so it is necessary to keep the temperature lower in the classroom, another change instituted in the school microsystem.

*The inspectors say: “I turn the air-conditioning on at maximum, for Valentina I turn it on at maximum”, the children are all wrapped up, freezing to death, and then Valentina is there, very happy, but they know it is for Valentina*. (P1)

*The room was prepared to receive him, even with air conditioning, because he can’t stand the heat either*. (P6)

Despite all the movement and adaptations in the school environment, unfortunately the condition of many children with EB is an impediment for their permanence and continuity in school.

*She used to attend (school), today she doesn’t anymore, because her hands are closed and she can’t sit for a long time, right? Everything is restricted for her. It’s very difficult*. (P3)

## The school-family mesosystem: possibilities to promote better school inclusion

Mothers experienced different feelings and emotions when starting the school trajectory of their child with EB. In this sense, one of the aspects that generated too much concern among the mothers was the risk of appearance of new injuries during the interaction between the students:

*I shouldn’t’ mention it, but I say it like this, children they don’t have much notion, so even without wanting to, they bump into each other, I was afraid they would hurt*. (P5)

The indignation at situations of prejudice and the wish that their children with EB were treated in a “normal” way was also present:

*I’ve cried a lot, I’ve fought. I fought with the principal (laughs), with the teacher. Because that’s how it is, you want your child to have a normal treatment* (P2)

*Furthermore, ambivalent feelings of pride and sadness at the mother-child separation were recalled: I took him away, he didn’t even look back. I cried and he stayed*. (P4)

However, close and shared relationships among the members of the mesosystem family-school favored the inclusion of children and adolescents with EB in school and attenuated the mothers’ negative feelings. From this perspective, by making information about the disease available to the families of the other classmates, the school successfully managed to bring together and involve children, families and professionals in the task of welcoming the child/adolescent with EB in the school environment.

*I enrolled Valentina and before she joined the class, the coordinator had a conversation at the parents’ meeting, the school also needed the help of these families to explain to their children at home, and if the children came home with some information, the families would also know how to answer these questions.* (P1)

The previous preparation of the child by the family microsystem also had a positive impact on school integration:

*Before he went to pre- school, I already paid a private teacher (…) so he already had this contact with school activity, with a teacher. So, going to school for him is fine, he isn’t seeing much difference* (P4).

A strategy adopted by mothers to ensure the well-being of the child during the stay at school is related to pain management*: I give him the pain medicine normally before going to school*. (P1)

Furthermore, the relationships established in the school-family mesosystem, sometimes conflictive and sometimes harmonious, reflected on structural and behavioral changes, as explained in the first category. Such achievements gave rise to contentment and satisfaction:

*The school, so, embraced him in a way, that we were very happy*.(P6)

Finally, even though advances have occurred, the school inclusion of a child/adolescent with EB is seen as a constant challenge for the family-school mesosystem: *So, it is a challenge for the school, it is a challenge for the family, it is a challenge for her*. (P1)

## DISCUSSION

The present research revealed the mothers’ perspective regarding the challenges in school inclusion of children/adolescents with EB as well as the benefits provided by this opportunity of social interaction. The school microsystem is one of the main contexts of human development for children and adolescents, besides the family, where they experience and build their identities in a more autonomous way^(15)^.

Studies have shown that attending school enhances the development and regulation of social behaviors, through opportunities for social and intellectual stimulation. Being out of school, on the other hand, has caused deleterous effects on mental health, well-being, and educational skills in children and adolescents^(18)^. Thus, the relationships established in this context are essential for the full development of this population, and should be seen by health professionals, considering the premises of the Health in the School Program^(13)^.

The beginning of the school career represented a stressful time for families, especially because of the reactions of peers, parents and educators. A similar situation has already been described in the literature where children/adolescents reported feeling avoided by their peers, due to physical differences^(3)^ or the false conception of being an infectious disease^(3, 5, 7)^. They were also frequently teased and experienced school bullying, with the use of offensive and derogatory terms^(3, 7)^. Besides the negative attitudes of other children who intentionally touched the skin of the child with EB to induce blisters^(3)^.

The lack of information and misconceptions about EB may represent a problem in the school environment^(5)^. Therefore, communication and education about EB favors understanding and reduces stigmatizing reactions in everyday situations, such as when inserting a child/adolescent with EB in a new school^(1, 9)^. In this direction, the role of health professionals is highlighted, especially nursing, to offer information about EB and other conditions, contributing to the preparation of the school and classrooms to receive the student with EB. In addition to the contributions of nursing, by offering instructions on the care of mucocutaneous lesions, dressings, medications and special accommodations for schools^(5)^.

Although a similar situation was not reported in the present investigation, it is important to discuss bullying and victimization by peers, because they have serious consequences for the mental health of children and adolescents with EB, such as depression and suicidal ideation^(10)^. Thus, it is essential to monitor children/adolescents with EB and question about the experience of teasing and the feeling of social isolation^(3)^. In this direction, the potential of nurses’ actions in preventing and/or facing bullying in schools is highlighted, based on interventions in the school-family mesosystem that encourage healthy behaviors, living with differences, promotion of quality of life, autonomy and emancipation, among other dimensions of care^(18, 19)^.

A study carried out with 50 Italian families of children with EB concluded the need for an interprofessional work to support families to identify and strengthen adaptive and coping behaviors^(20)^. Again, due to the privileged place of Nursing in the different points of care of the network, this professional is essential in this debate and practice.

Another important aspect presented in the results concerns the need to affirm that the child/adolescent does not present learning problems due to EB. There is no scientific evidence to support this association^(3, 5)^. Individuals with EB may miss school days due to medical appointments, hospitalizations, sick days and even school bullying^(21)^. School absenteeism can have a significant impact on the learning process, and may contribute to lower academic performance, which can be mistakenly confused with cognitive deficit^(3)^. Also, the extent of the pain and physical discomfort caused by EB must be recognized. The agony experienced impairs or prevents the child/adolescent from maintaining focus on school activities^(9)^. It is essential for educators and peers to create an atmosphere of inclusion in a safe environment for the child/adolescent with EB^(3)^.

In order to promote the inclusion of children/adolescents with EB, the school microsystem has promoted adaptations of the teaching-learning process, structural changes, and hiring of caregivers. The literature corroborates by stating the significant dependence of this group of children/adolescents and the need for human and structural resources to ensure their quality of life^(3)^.

In the present study, the experience of an adolescent who felt uncomfortable with the constant surveillance by the caregiver and wished for more independence at school was portrayed. Thus, it is proposed here the reflection of the need for a balance between these measures, so that they do not negatively impact the relationships of children/adolescents with EB established in the school microsystem. The restrictions in participating in social activities that can cause damage to the skin, such as the practice of sports, can cause frustration^(5)^. Many times, excessive care makes children and adolescents with EB feel “inside a bubble”, keeping them “excluded”, for fear of getting hurt in moments of interaction with peers and in games^(3)^.

As for the limitations of this study, we highlight the unfeasibility of carrying out the interviews in person, as well as not having analyzed the perspective of the other members of the school-family mesosystem, such as the child/adolescent with EB, his/her peers, teachers and family members of other children. However, because it is a rare clinical condition, remote interviews aggregated experiences from different Brazilian localities, allowing us to explore different contexts and realities.

The study showed the relevance of health education, orientation and sensitization of school professionals, parents and children/adolescents about EB, providing a better experience in the school inclusion of these CSHCN. In this direction, the educator role of nurses and their work in Primary Health Care and in schools is highlighted, with contributions in the development of strategic actions for the adaptation, protection, and care of these children/adolescents in the school environment, confronting bullying and social inclusion.

## CONCLUSION

The process of school inclusion of children and adolescents with EB is permeated by challenges and an intensity of feelings, fears, and anguish in mothers, due to the stigma of the disease, lack of knowledge, and the false conception of it as an infectious-contagious condition, promoting the social isolation of the child/adolescent with EB, in addition to the unexpected reaction from peers, teachers, and parents. It was evidenced that the previous preparation of the school, with meetings with parents, teachers and other children/adolescents with the offer of information in appropriate language, provided the development of a welcoming and receptive atmosphere. Also, the presence of a full support professional, the relevance of structural and environmental adaptations for the prevention of mucocutaneous lesions in the school environment, and the guarantees of comfort and protection conditions for the child/adolescent with EB were highlighted as essential and strengthening elements in this process. The school represented an environment of enriching experiences and social inclusion, providing the creation of new ways of interaction with peers and teaching-learning, considering the needs and specificities of children and adolescents with EB.
